# The RNA binding protein Quaking represses splicing of the Fibronectin EDA exon and downregulates the interferon response

**DOI:** 10.1093/nar/gkab732

**Published:** 2021-08-24

**Authors:** Kuo-Chieh Liao, Vanessa Chuo, W Samuel Fagg, Cassandra M Modahl, Steven Widen, Mariano A Garcia-Blanco

**Affiliations:** Programme in Emerging Infectious Diseases, Duke-NUS Medical School, Singapore 169857, Singapore; Programme in Emerging Infectious Diseases, Duke-NUS Medical School, Singapore 169857, Singapore; Transplant Division, Department of Surgery, University of Texas Medical Branch, Galveston, TX 77555, USA; Department of Biochemistry and Molecular Biology, University of Texas Medical Branch, Galveston, TX 77555, USA; Department of Biological Sciences, National University of Singapore, Singapore 119077, Singapore; Department of Biochemistry and Molecular Biology, University of Texas Medical Branch, Galveston, TX 77555, USA; Programme in Emerging Infectious Diseases, Duke-NUS Medical School, Singapore 169857, Singapore; Department of Biochemistry and Molecular Biology, University of Texas Medical Branch, Galveston, TX 77555, USA; Department of Internal Medicine, University of Texas Medical Branch, Galveston, TX 77555, USA; Institute for Human Infections and Immunity, University of Texas Medical Branch, Galveston, TX 77555, USA

## Abstract

Quaking (QKI) controls RNA metabolism in many biological processes including innate immunity, where its roles remain incompletely understood. To illuminate these roles, we performed genome scale transcriptome profiling in QKI knockout cells with or without poly(I:C) transfection, a double-stranded RNA analog that mimics viral infection. Analysis of RNA-sequencing data shows that QKI knockout upregulates genes induced by interferons, suggesting that QKI is an immune suppressor. Furthermore, differential splicing analysis shows that QKI primarily controls cassette exons, and among these events, we noted that QKI silences splicing of the extra domain A (EDA) exon in fibronectin (FN1) transcripts. QKI knockout results in elevated production and secretion of FN1-EDA protein, which is a known activator of interferons. Consistent with an upregulation of the interferon response in QKI knockout cells, our results show reduced production of dengue virus-2 and Japanese encephalitis virus in these cells. In conclusion, we demonstrate that QKI downregulates the interferon system and attenuates the antiviral state.

## INTRODUCTION

Effective immune responses are tightly controlled to ensure protective immunity without causing deleterious effect to the host system. This control is particularly important for innate immunity and the type I interferon system, as they serve as the first line of defense against a myriad of invading viruses. To achieve this level of control, many regulatory mechanisms are in place at transcriptional and post-transcriptional levels (1–3), including alternative splicing. Alternative splicing is a highly regulated process that enables single genes to generate multiple distinct mRNAs that can encode distinct proteins (4,5). This process provides a critical layer of gene regulation in higher eukaryotic cells and expands proteome complexity. Although this expansion in diversity can provide advantages to immune systems, dysregulation of alternative splicing can result in malfunctioning of the immune system and autoimmune diseases (6,7). Alternative splicing is primarily executed by the action of regulatory RNA-binding proteins (RBPs) on the core splicing machinery, a ribonucleoprotein (RNP) called the spliceosome, which involves dynamic RNA-RNA, RNA-protein, and protein-protein interactions (8).

Quaking (QKI) is a member of the signal transduction and activator of RNA (STAR) family of hnRNP K homology (KH)-type RBPs and is involved in many aspects of RNA metabolism, including splicing (9,10). Three alternatively spliced QKI isoforms (QKI-5, QKI-6 and QKI-7) are produced from a single gene (11,12) with QKI-5 predominantly residing in the nucleus (13). QKI function is primarily executed by binding to RNAs via a specific sequence motif (core site: AC/UUAA; ‘half site’: UAAY) (14–16). Aberrant alteration of QKI function has been implicated in several human diseases (17–20). Diverse roles have been described for QKI in immunity: QKI plays pro-viral roles in promoting replication of Herpesvirus type 1 (21) and Zika virus (ZIKV) (22), but plays anti-viral roles in restricting replication of a dengue virus serotype 4 (DENV4) clinical isolate (23). From the perspective of the host, QKI was shown to dampen bacterial lipopolysaccharide (LPS)-induced inflammation and suppress the interferon (IFN) response (24–26). Despite these recent advances, the function of QKI in shaping host immunity remains incompletely understood.

Here, we performed genome scale transcriptome profiling in HuH7 wildtype (WT) and QKI knock-out (QKO) cells in the absence and presence of poly(I:C) transfection. Poly(I:C) is a double-stranded RNA analog that mimics the effects of viral infection. Analysis of RNA-seq data revealed thousands of differentially expressed genes (DEGs) with elevated transcript levels observed from many interferon (IFN)-related and pro-inflammatory genes in QKO cells, suggesting an immune suppressive role for QKI. Additionally, hundreds of differential splicing events between WT and QKO cells were identified, including in the Fibronectin (*FN1*) gene product. Multiple isoforms are generated from this gene by alternative splicing (27,28), and spliced variants which retain the extra domain A (EDA) exon have previously been associated with several human diseases and immune activation (28–31). We observed that QKI-5 binds to the intron upstream of the EDA exon and blocks EDA exon inclusion in FN1. Increased EDA inclusion was also observed in several other different QKI-deficient human cell types. This resulted in elevated secretion of the FN1-EDA protein isoform, which can contribute to systemic inflammation and immune responses. Lastly, we showed that QKO cells produce lower levels of progeny dengue virus-2 and Japanese encephalitis virus than WT cells, altogether suggesting that QKI suppresses IFN action and antiviral immunity.

## MATERIALS AND METHODS

### Cell lines

HuH7 and A549 cells were maintained in Dulbecco's modified Eagle's medium (DMEM, Gibco) supplemented with 10% fetal bovine serum (FBS), 1% penicillin–streptomycin (Pen Strep, Gibco), and 1% HEPES (Gibco) in a 37°C humidified incubator with 5% CO_2_. QKI knock-out cell lines (HuH7 QKO#3, HuH7 QKO#13, HuH7 QKO#14 and A549 QKO#1) were generated using CRISPR–Cas9 technology as previously described (26). Guide sequences and editing events are listed in Supplementary Table S3. HuH7 QKO#3 cells stably expressing QKI-5 (Q5B) were maintained in regular media supplemented with additional geneticin (1mg/ml). Additionally, mouse cells (MEF and NIH/3T3) were maintained in DMEM supplemented with 10% FBS, 1% Pen Strep and 1% HEPES.

### RNA sequencing and bioinformatic data analyses

HuH7 WT and QKO#3 cells were seeded at 5 × 10^5^ cells per well in six-well plates the day before stimulation. Cells were transfected with 1.5 μg poly(I:C) HMW (tlrl-pic; InvivoGen) using Lipofectamine 2000 (Invitrogen). At 4 h post-transfection, cells were rinsed twice with PBS and replaced with fresh media. Cells were harvested at nine hours post transfection for subsequent RNA isolation using RNAzol RT (Molecular Research Center). After removing genomic DNA via the RapidOut DNA removal kit (Thermo Fisher Scientific), RNA samples were re-purified using RNAzol RT. Total RNA (∼0.5 ug) was then used as template to generate libraries using the Illumina Stranded Total RNA Prep with Ribo-Zero Plus kit as recommended by the manufacturer. Library quality was determined by Agilent Bioanalyzer analysis and quantity was determined by RT-qPCR. All libraries were uniquely indexed and combined into one pool, which was run on two NextSeq 550 High Output 75 base paired-end runs following the manufacturer's recommendations. The fastq files from the two runs were combined and were checked for quality using FastQC (32) before further analysis.

Splicing analysis was carried out using *vast-tools* program version 2.2.2 (33) by aligning the paired-end reads to the vast-tools human database (vastdb.hsa.16.02.18) using the default parameters. Three replicates of each condition were compared with the diff function of vast-tools to determine differential splicing. Two parameters were used to establish differential splicing events: E [DPsi], which refers to the difference in splicing between the experimental conditions, and max(x)@P(jDPsij >x)>0.95, which indicates the change in splicing at 95% confidence level. Psiplot and Pheatmap packages were used to produce heatmaps in R. For differential gene expression analysis, Salmon version 0.14.1 (34) was used to quantify transcript-level abundance from RNA-seq data and differential gene expression analysis was performed using DEseq2 version 1.24.0 (35). Gene set enrichment analysis (GSEA) was conducted using the GSEA software (36).

### Validation of differential gene expression and differential splicing events

Total RNA was extracted via the EZNA Total RNA Kit I (OMEGA bio-tek), and RapidOut DNA removal kit was used to remove genomic DNA (Thermo Fisher Scientific). For validation of differential gene expression, reverse transcription was performed on 1 μg total RNA using the iScript cDNA synthesis kit (Bio-Rad), and real-time PCR (RT-qPCR) was performed using SsoAdvanced Universal SYBR Green Supermix reagent (Bio-Rad) using manufacturer's instructions. For validation of differential splicing events, reverse transcription was performed on 1μg total RNA using the SuperScript III First-Strand Synthesis System (Invitrogen). Subsequently, RT-PCR was performed using Q5 High-Fidelity 2X Master Mix (NEB) with the following program: 98°C for 30 s, followed by 25 cycles at 98°C for 10 s, 55°C for 20 s and 72°C for 30 s, and a final step of 72°C for 2 min. Primers for both RT-qPCR and RT-PCR are listed in Supplementary Table S5. Amplified PCR products were loaded in 5% TAE-acrylamide gels for electrophoresis. For CFH gene splicing analysis, the thermocycling 72°C extension time was increased to 1minute and resultant PCR products were separated using 1% agarose gel. Bands were visualized in a UV light transilluminator (Bio-Rad), and densitometry analysis of PCR fragments corresponding to inclusion and skipping events was performed using ImageJ software. In addition, representative PCR products, except CFH, were further analyzed in DNA 1000 chips (Agilent) on the Agilent 2100 Bioanalyzer.

### Small interfering RNA (siRNA) transfection

HuH7 WT and QKO#3 cells were transfected with two rounds of 20 nM non-targeting siCtrl (AllStars Negative, Qiagen) or siRNA targeting FN1-EDA (siEDA – CAU UGA UCG CCC UAA AGG A dTdT)(37) using Lipofectamine RNAiMax (Invitrogen) with manufacturer's protocol. Cells were harvested for immunoblotting analysis two days after the second transfection. MEF and NIH/3T3 cells were seeded at 5 × 10^4^ cells per well in 12-well plates and reverse-transfected with 50 nM non-targeting siCtrl or siRNA targeting QKI (siQk - GAC GAA GAA AUU AGC AGA GUA UU) using Lipofectamine RNAiMax. At two days post transfection, protein expression was assessed by immunoblotting.

### Western blotting

HuH7 WT and QKO#3 cells, along with A549 WT and QKO#1 cells, were seeded at 1 × 10^5^ cells per well in 12-well plates overnight. After rinsing twice with PBS, cells were incubated with 300 μl serum-free media. On the following day, conditioned media was collected from each well and spun down at 14 000 rpm for 5minutes at 4°C. Supernatants were transferred to new tubes for subsequent detection of proteins by immunoblotting. Cells were rinsed once with PBS before lysing in RIPA buffer (Santa Cruz Biotechnology) for further processing in western blotting. Roche phosphatase inhibitor was added in RIPA buffer for probing phosphorylated proteins. For probing FN1 and FN1-EDA in both cells and supernatants, samples were mixed with SDS loading dye without 2-mercaptoethanol and were loaded in 4–15% polyacrylamide gels (Bio-Rad) for electrophoresis. After electrophoresis, samples were transferred to PVDF membranes (Bio-Rad), blots were blocked in PBST (0.5% Tween-20) with 5% blotting grade blocker (Bio-Rad). Blots were then washed and incubated at 4**°**C overnight with the following primary antibodies: rabbit QKI-5 antibody (A300-183A; Bethyl Laboratories), mouse fibronectin-EDA (FN1-EDA) antibody (ab6328; Abcam), mouse fibronectin (FN1) antibody (sc-8422; Santa Cruz), rabbit alpha-fetoprotein (AFP) antibody (4448; Cell Signaling), mouse pan-QKI antibody (clone N147/6; UC Davis/NIH NeuroMab Facility), mouse FSCN1 antibody (sc-46675; Santa Cruz), rabbit phospho-Akt antibody (4060; Cell Signaling), mouse Akt antibody (2920; Cell Signaling) and mouse actin antibody (MAS-11869; Thermo Fisher Scientific). Goat anti-mouse HRP (115-035-003; Jackson ImmunoResearch) and goat anti-rabbit HRP (111-035-003; Jackson ImmunoResearch) were used to visualize blots on a chemiluminescence imaging system (Bio-Rad). Densitometric analysis was performed using ImageJ software.

### RNA immunoprecipitation (RIP)

HuH7 QKO#3 cells complemented with FLAG-tagged QKI-5 expression were seeded around 2 × 10^6^ cells in 10cm dishes. One day post seeding, cells were harvested, pelleted, and lysed in a buffer volume roughly equivalent to the cell pellet volume of RIP lysis buffer (200 mM KCl, 20 mM HEPES pH 7.2, 2% *N*-dodecyl-β-d-maltoside, 1% Igepal CA-360, 100 U/ml Murine RNase inhibitor [NEB]). Subsequent lysates were cleared by centrifugation, and protein was normalized across samples to about 200 μg per RIP reaction. To prepare RIP assay beads, Dynabeads protein G (Invitrogen) were blocked with BSA on the day before cell harvest and then incubated with 5 μg of mouse IgG control antibody (12-371; Millipore) or 5μg FLAG antibody (F3165; Sigma) with head-to-tail rotation at 4°C overnight. Antibody-coupled beads were washed three times with RIP assay buffer (50 mM Tris–HCl pH 7.5, 150 mM NaCl, 1 mM MgCl_2_ and 0.05% Igepal CA-360) and subsequently incubated with the prepared lysates on rotation at 4°C for 1 h. Complexes were washed four times in RIP assay buffer, and immunoprecipitated protein and RNA (extracted by RNAzol^®^ RT (Molecular Research Center) or TRIzol reagent) were analyzed by immunoblotting and RT-qPCR, respectively. Primer sequences are listed in the Supplementary Table S5.

### Viral infection and plaque assays

Low-passage SA 14-14-2 Japanese encephalitis virus (JEV) was provided by Dr Ashley L. St. John and propagated as described previously (38). Dengue 2 virus (DENV2) was initially isolated from patient sera and propagated as described previously (39). JEV and DENV2 infections of HuH7 cells were carried out at MOIs of 0.5 and 10, respectively. At 24 h after JEV infection, supernatants were collected for titer determination using plaque assay. Regarding DENV2, supernatants were collected at 48hours post-infection. For plaque assay, BHK-21 cells were seeded at 1.7 × 10^4^–1 × 10^5^ cells per well in 24-well plates the day prior to infection. Supernatant media from previously infected HuH7 cells were serially diluted by 10-fold in serum-free RPMI 1640 media. After removing media from the BHK-21 cells, the monolayers were incubated with inoculum for 1hour. Inoculum was then substituted with overlay media containing RPMI 1640, 2% FBS and 1% Aquacide II (Calbiochem). Fixation was performed by adding 10% formaldehyde-PBS directly above overlay media for 1hour. For JEV titration, cells were fixed three days post infection. For DENV2 titration, cells were fixed 5 days post infection. After disposing overlay media/fixing solution, plates were rinsed and stained with 1% crystal violet solution for five minutes to reveal plaques.

## RESULTS

### Transcriptome profiling reveals that QKI represses the interferon response

**Figure 1.**
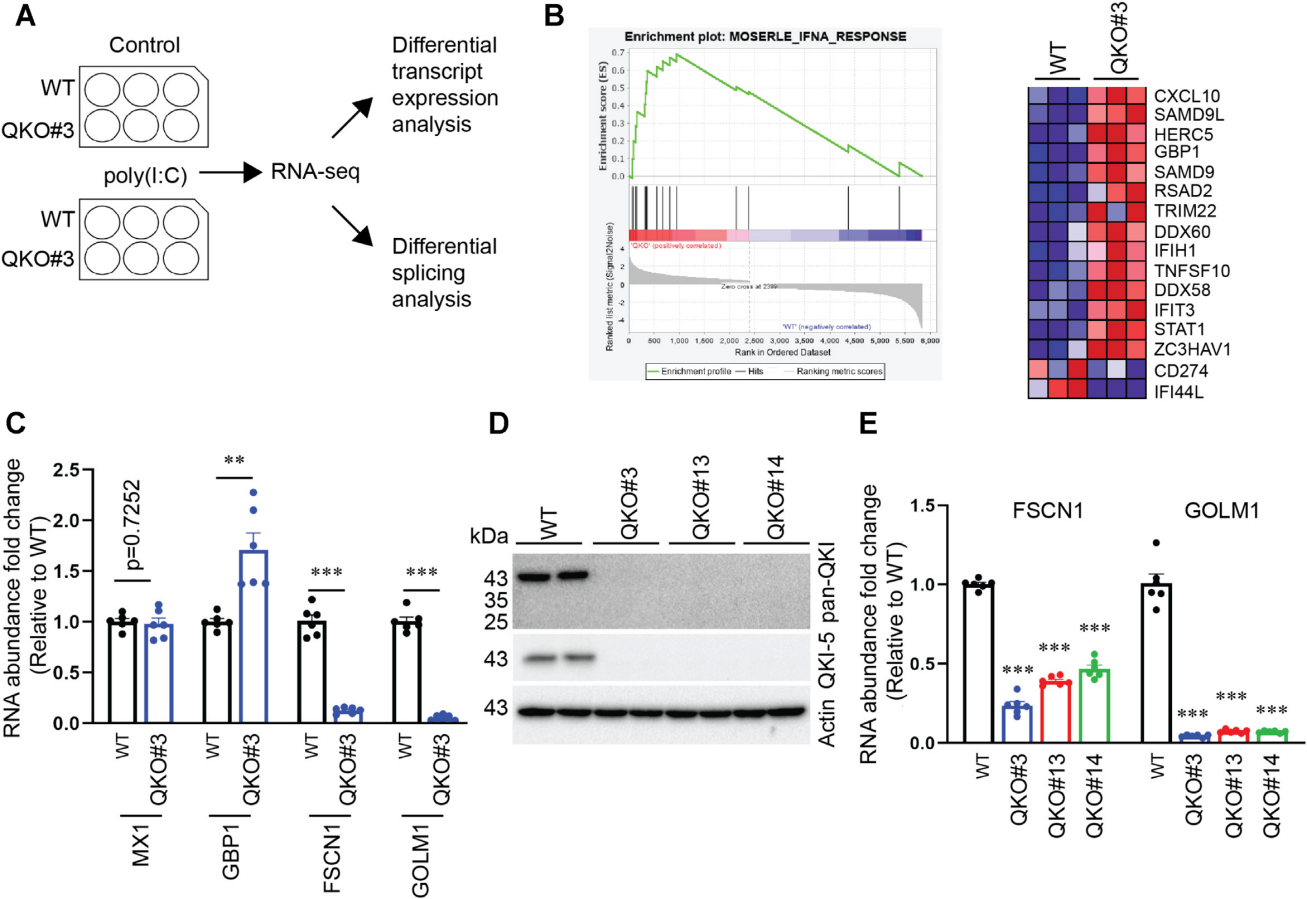
Transcriptome profiling of WT and QKO#3 cells reveals QKI-dependent genes in IFN pathways. (**A**) Schematic illustration of experimental design. (**B**) Enrichment plot and heatmap from the comparison with gene set ‘MOSERLE_INFA_RESPONSE’. Red and blue indicate up-regulated and down-regulated trends respectively. (**C**) HuH7 WT and QKO#3 cell lysates were harvested and RNA abundance of indicated genes were measured by RT-qPCR. (**D**) Representative immunoblotting results showing expression level of QKI in WT and various QKO cells. (**E**) RNA abundance of *FSCN1* and *GOLM1* in HuH7 WT and QKO cells by RT-qPCR. The RT-qPCR data were reported relative to the WT. Data are mean ± SEM from two independent experiments, and each experiment had three wells that were treated independently (replicates = 6). Each dot represents one biological replicate. Statistical significance was determined using a two-tailed *t* test: **P < 0.01; ***P < 0.001.

To investigate the mechanisms by which QKI regulates host immunity, we performed genome scale transcriptome profiling in parental (WT) and QKI knock-out (QKO#3) HuH7 cells (26) activated by poly(I:C) transfection (Figure 1A). RNA-seq data, which had an average depth of 75M reads per sample, were analyzed to identify differential expression and processing of transcripts. Of note, principal component analysis (PCA) of differentially expressed genes (DEGs) showed evident clusters for each condition, suggesting reproducibility between three biological replicates for each condition (Supplementary Figure S1A).

To identify DEGs between conditions, we used Salmon (34) to quantify transcript abundance and DESeq2 (35) to determine differential gene expression. Among the thousands of DEGs identified between WT and QKO#3 cells (adjusted *P* value < 0.05), some were observed only in untreated cells, some only in poly(I:C)-treated cells and some in both conditions (Supplementary Table S1, Supplementary Figure S1B). Several changes impacted genes with well-characterized roles in innate immunity and inflammation (Supplementary Figure S1B). Expression of IL6 (40), for instance, is activated by poly(I:C) and was found to be more than 2-fold higher in poly(I:C)-activated QKO#3 cells (Supplementary Figure S1C). Although QKI KO did not globally alter the gene expression changes induced by poly(I:C) treatment, a few poly(I:C)-induced events were sensitive to QKI status (Supplementary Figure S1D). These observations, taken together, led us to hypothesize that QKI can play a role in regulating innate immune responses.

We further investigated this hypothesis using Gene Set Enrichment Analysis (GSEA) (36), which permits the comparison of gene sets, which are composed of genes up- or down-regulated under specific conditions, to our QKI KO data. Several GSEA gene sets associated with IFN responses were enriched for genes overexpressed in QKO#3 cells relative to WT cells (Supplementary Table S2). For example, the top scoring REACTOME_INTERFERON_GAMMA_SIGNALING (41,42) gene set includes many IFN and immune-related genes that are significantly up-regulated in the QKO#3 cells (Supplementary Figure S1E). These data indicate that QKI negatively regulates IFN responses. To more stringently test this, we analyzed data comparing WT and QKO#3 cells that had not been treated with poly(I:C). Among GSEA gene sets that significantly correlated with gene expression changes caused by QKI KO in the absence of poly(I:C) transfection, the second highest scoring was the MOSERLE_IFNA_RESPONSE gene set (43) (normalized enrichment score of 2.42 with FDR *q*-val = 0.002; Figure 1B, Supplementary Table S2). This analysis implied that QKI represses the expression of many genes induced by type I IFNs, which is consistent with our previous report that QKI suppresses IFN induction (26).

We selected four genes with known functions in the IFN response for validation of the RNA-seq results using real-time quantitative PCR (RT-qPCR). For RT-qPCR, relative levels of gene expression were normalized to the geometric mean of two reference genes, *SDHA and HPRT1*, and are expressed relative to the untransfected WT control. While the IFN stimulated gene (ISG) *MX1* was not affected by QKI KO in the absence of poly(I:C) treatment, expression of another ISG *GBP1* was consistently increased (Figure 1C). QKI KO profoundly reduces the expression of *FSCN1* and *GOLM1* (Figure 1C), which have been reported to negatively regulate IFN response via distinct mechanisms (44,45). To further test if QKI promotes *FSCN1* and *GOLM1* expression, we measured their transcript levels in two additional QKO cells, QKO#13 and QKO#14 (Figure 1D and Supplementary Table S3). Each of the three independently-derived HuH7 QKO cells showed a significant reduction in *FSCN1* and *GOLM1* RNA levels (Figure 1E). FSCN1 protein was also significantly reduced in all QKI-deficient cells (Supplementary Figure S2A).

To assess the mechanism through which QKI promoted FSCN1 and GOLM1 expression, we reanalyzed QKI eCLIP data from ENCODE (46). While no QKI eCLIP peaks were observed mapping to GOLM1 transcripts, we found evidence of QKI binding FSCN1 RNAs, including several sites in the 3′ UTR (Supplementary Figure S2B) This observation suggested that QKI might regulate *FSCN1* expression directly via binding its 3′ UTR to promote transcript stability. To test this hypothesis, we constructed a Firefly luciferase (F Luc) reporter plasmid including the FSCN1 3′ UTR sequence and transfected it into WT and QKO#3 cells along with Renilla luciferase (R Luc) plasmids as a transfection control. In QKO#3 cells, a reduction of ∼25% relative luciferase activity was observed, suggesting that QKI promotes FSCN1 expression through its 3′ UTR (Supplementary Figure S2C).

The effects of QKO on *GBP1* and *MX1* are consistent with our previous report (26) but also suggest that QKI selectively regulates certain pathways induced by IFNs. The data on *FSCN1* and *GOLM1* support our previous findings that QKI moderates the IFN response by interfering with MAVS signalling (26). We previously showed that QKI down-regulates MAVS protein levels and here we show that QKI up-regulates *FSCN1* and *GOLM1*, both of which are known inhibitors of MAVS signaling (Supplementary Figure S2D). Thus, the current findings add an additional layer to the complexity of the QKI-regulated IFN response.

### QKI and poly(I:C) modulate orthogonal alternative splicing programs

**Figure 2.**
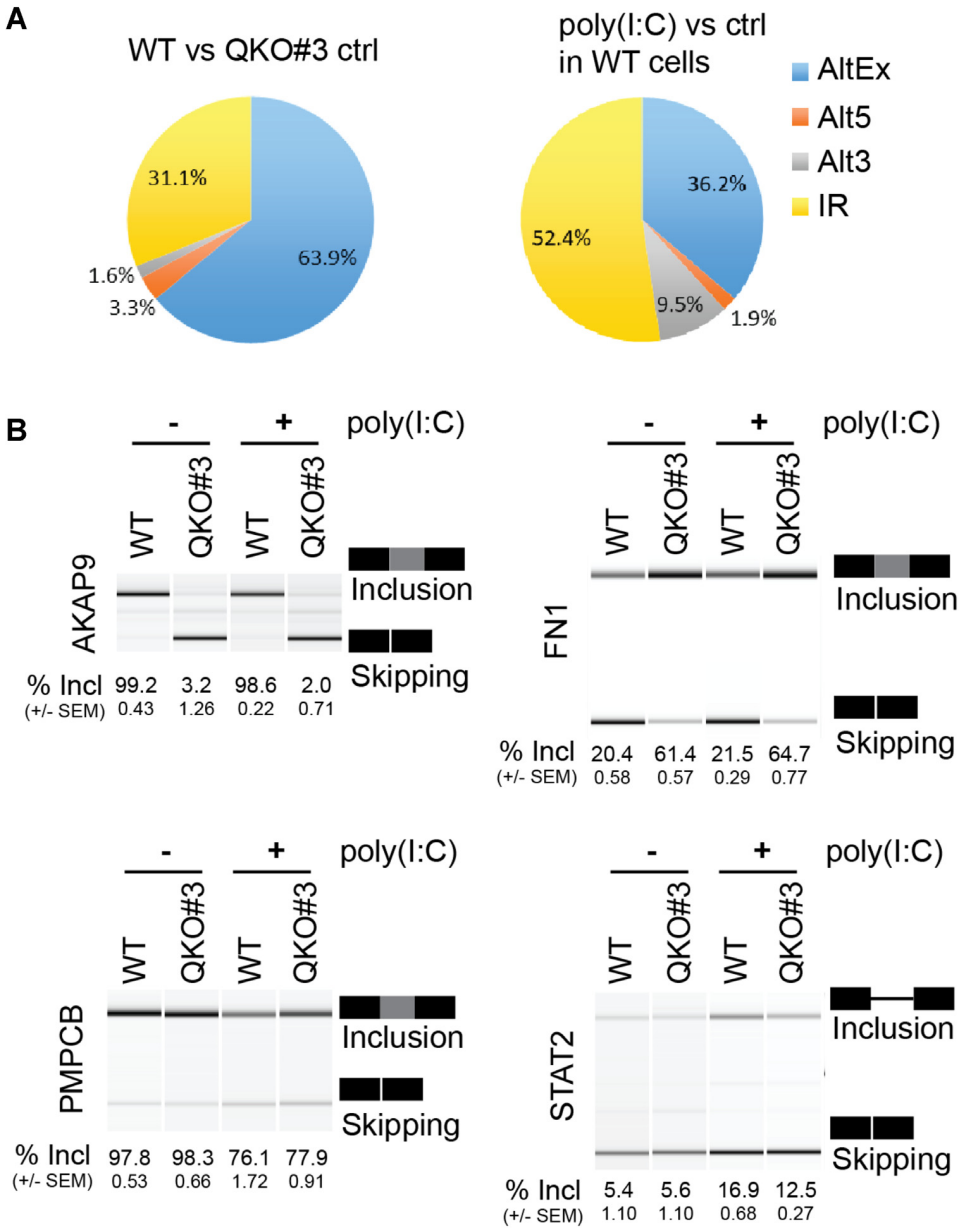
QKI and poly(I:C) regulate orthogonal splicing programs. (**A**) Pie charts showing different types of differential splicing events. (**B**) HuH7 WT and QKO#3 cells were left untreated or transfected with 1.5 μg poly(I:C). At nine hours post transfection, lysates were harvested. After cellular RNA isolation, RT-PCR was performed to amplify splicing variants of indicated genes. PCR products were separated on 5% TAE-acrylamide gels and imaged in a UV light transilluminator or analyzed using the Agilent 2100 Bioanalyzer. Representative data were shown. The ratio of inclusion to skipping was recorded as a percent of sequence inclusion (% Incl). Data are mean ± SEM from two independent experiments, and each experiment had three wells that were treated independently (replicates = 6). Exon/intron genomic coordinates are provided in Supplementary Table S4.

Since QKI plays critical roles in regulating alternative splicing (9,10,47), we analyzed our RNA-seq data for transcript isoform changes in the four conditions tested. The program *vast-tools* (33,48) predicted 122 differential splicing events between WT and QKO#3 cells (Supplementary Table S4), the majority (63.9%) of which are cassette exon skipping events (AltEx) (Figure 2A). Thirty-eight events are intron retention (IR), and fewer than ten events are predicted to involve alternative 5′ or 3′ splicing sites (Alt5 and Alt3). The QKI-dependent AltEx and IR events, are approximately equally divided in terms of the direction of the effect (Supplementary Table S4). A comparison of significantly changing splicing events with another dataset obtained by QKI knockdown (47) showed sixteen common events (Supplementary Table S4). In the presence of poly(I:C) transfection, there are 109 differential splicing events between WT and QKO#3 cells (Supplementary Table S4). Similar to the poly(I:C) untransfected condition, more than half of these 109 differential splicing events are AltEx. There are 42 QKI-dependent alternative isoform changes that are seen in both poly(I:C)-treated and untreated cells (Supplementary Figure S3A). These data confirm the work of others (9,10,47) who have shown that QKI is an important splicing regulator.

Unlike QKI-regulated splicing events, more than half of poly(I:C)-activated splicing changes involve intron retention (IR) (Figure 2A). As noted above for transcript level changes, alternative isoform changes induced by poly(I:C) were not globally disrupted by QKI KO (Supplementary Figure S3B). This predominantly orthogonal regulation of alternative splicing by QKI and poly(I:C) was confirmed through RT-PCR validation of cassette exon and intron retention changes predicted by vast-tools. QKI KO led to exon skipping in *AKAP9* and *INSR*, and exon inclusion in *FN1*, *CTNND1* and *ADD3*, independent of poly(I:C) treatment (Figure 2B and Supplementary S3C). Upon poly(I:C) transfection, however, *PMPCB* and *EIF4A2* exhibited increased inclusion of an alternative exon and *STAT2* and *CFH* displayed increased intron retention, independent of QKI presence or absence (Figure 2B and Supplementary Figure S3C). An exception to this orthogonal behavior was noted in *CLK1* transcripts where QKI had opposite, albeit low-magnitude, effects on cassette exon inclusion in untreated vs poly(I:C) treated cells (Supplementary Figure S3C).

Although QKI does not appear to exert widespread control on the alternative splicing program induced by poly(I:C) treatment, we noted that QKI promotes skipping of the pro-immune *FN1* extra domain A (EDA) exon (Figure 2B) (28,31,49). The repressive effect exerted on the EDA exon, together with the observed positive regulatory effects on *FSCN1* and *GOLM1* expression, suggest a model in which QKI suppresses IFN activation via various pathways (Supplementary Figure S3D).

### QKI-5 represses EDA exon inclusion in FN1

**Figure 3.**
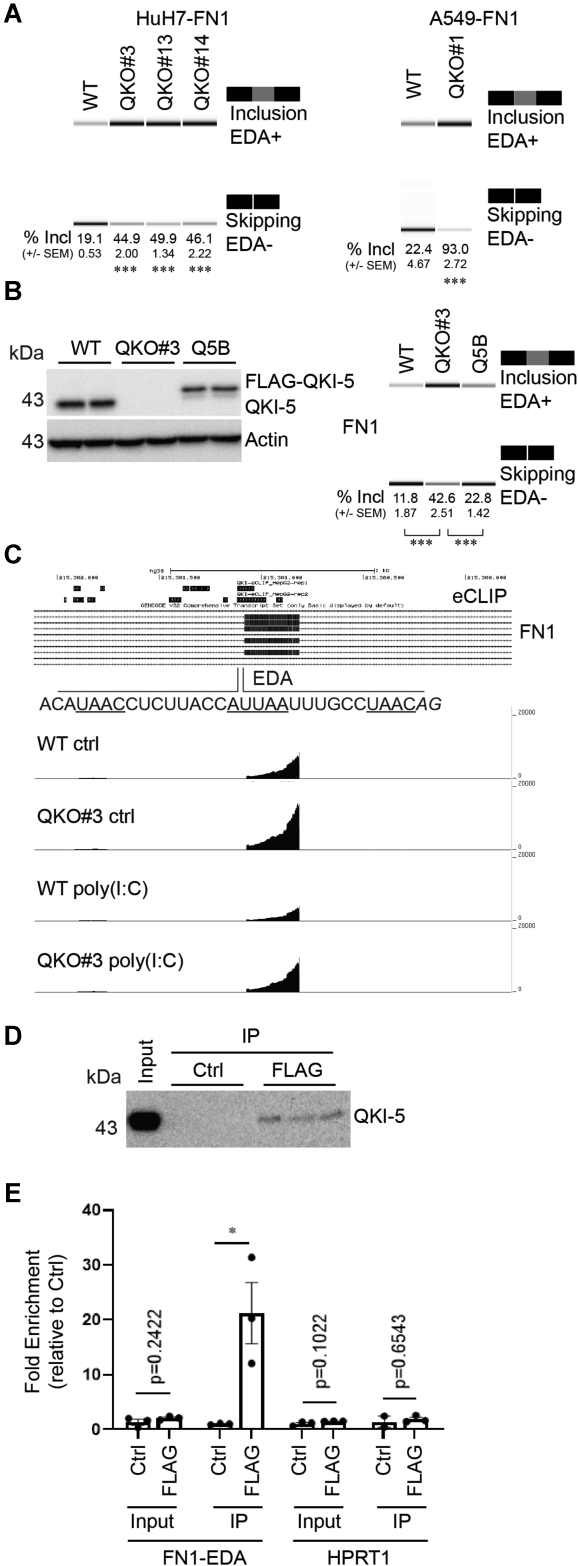
QKI-5 promotes FN1 EDA exon skipping. (**A**) HuH7 WT and three QKO cells (QKO#3, QKO#13 and QKO#14), in addition to A549 WT and QKO#1 cells, were seeded overnight. FN1 splicing variants were amplified from cellular RNA via RT-PCR. PCR products were separated on 5% TAE-acrylamide gels and percent of EDA sequence inclusion (% Incl) in FN1 was reported. Representative images from Agilent 2100 Bioanalyzer were shown. (**B**) HuH7 WT, QKO#3 and Q5B cells were seeded overnight before processing for RT-PCR analysis as mentioned above. Percent of EDA inclusion events (% Incl) in FN1 was recorded. (**C**) eCLIP data showing regions bound to QKI-5. Tracks showing the transcript abundance of EDA exon in *FN1* gene in WT and QKO#3 cells with or without poly(I:C). (**D**) Representative blot showing immunoprecipitated QKI-5. (**E**) RT-qPCR analysis of input and immunoprecipitated samples using primers targeting FN1-EDA and HPRT1. Data are mean ± S.E.M from at least two independent experiments, and each experiment has two or three wells that were treated independently (replicates = 5 or 6). Statistical significance was determined using a two-tailed *t* test: *P < 0.05; ***P < 0.001.

To confirm that QKI represses EDA exon inclusion, we evaluated *FN1* EDA exon alternative splicing in several independent QKI-deficient cells. In agreement with our finding in QKO#3 cells, increased EDA exon inclusion was also observed in HuH7 QKO#13 and QKO#14 cells and in QKI-deficient A549 QKO#1 cells (Figure 3A) (Supplementary Table S3). To determine if the QKI-5 isoform is sufficient to promote EDA exon skipping, we restored QKI-5 expression in QKO#3 cells (Q5B in Figure 3B). QKI-5 was selected because it is the primary isoform expressed in HuH7 cells (Figure 1D) and is necessary and sufficient for splicing function (50). As expected, rescuing with FLAG-QKI-5 restored skipping of the EDA exon in QKO#3 cells (Figure 3B), therefore QKI is necessary and QKI-5 is sufficient for EDA exon skipping. These data establish QKI as a potent repressor of EDA exon inclusion in *FN1* primary transcripts.

We next addressed whether or not QKI regulates the *FN1* EDA exon directly. We reanalyzed QKI-5 eCLIP data from ENCODE (46) and observed QKI binding primarily in the intron upstream of the EDA exon (Figure 3C). These data indicate that QKI binds sequence motifs in the intron upstream of the EDA exon and are consistent with previous observations that QKI promotes exon skipping by binding to the upstream intron (9,50). Consistent with the eCLIP data, we noted the presence of several putative QKI binding motifs in the intron upstream of EDA (core site: AUUAA and half site: UAAY) (15,16). Importantly, RNA immunoprecipitation (RIP) experiments show an enrichment of FN1-EDA in QKI-5 immunoprecipitated complexes, supporting that there is an association between QKI-5 and *FN1*-EDA transcripts in this system (Figure 3D and E). These experiments suggest that QKI regulates the *FN1* EDA exon directly.

Since both QKI and alternative splicing of the EDA exon are conserved in many bony vertebrates (euteleostomes), we wondered if QKI-regulated EDA alternative splicing is evolutionarily conserved. To address this, we examined intronic sequences upstream of the EDA exon in several vertebrate species and noted the presence of putative QKI binding motifs in the same region as observed in the human genome, suggesting that direct regulation by QKI is likely to be conserved (Supplementary Figure S4A). We also addressed conservation directly by knocking down Qk (the mouse homolog of human QKI) in mouse embryonic fibroblasts (MEF) and mouse fibroblast cells (NIH/3T3). We observed that Qk knockdown efficiently reduced Qk-5 expression in both MEF and NIH/3T3 cells (Supplementary Figure S4B), and this reduction in Qk levels resulted in an increase of EDA exon inclusion in *Fn1* (Supplementary Figure S4C). We conclude that the direct regulation of EDA exon inclusion by QKI is conserved in mice and humans, and likely in all vertebrates.

### FN1-EDA protein expression increases in the absence of QKI

**Figure 4.**
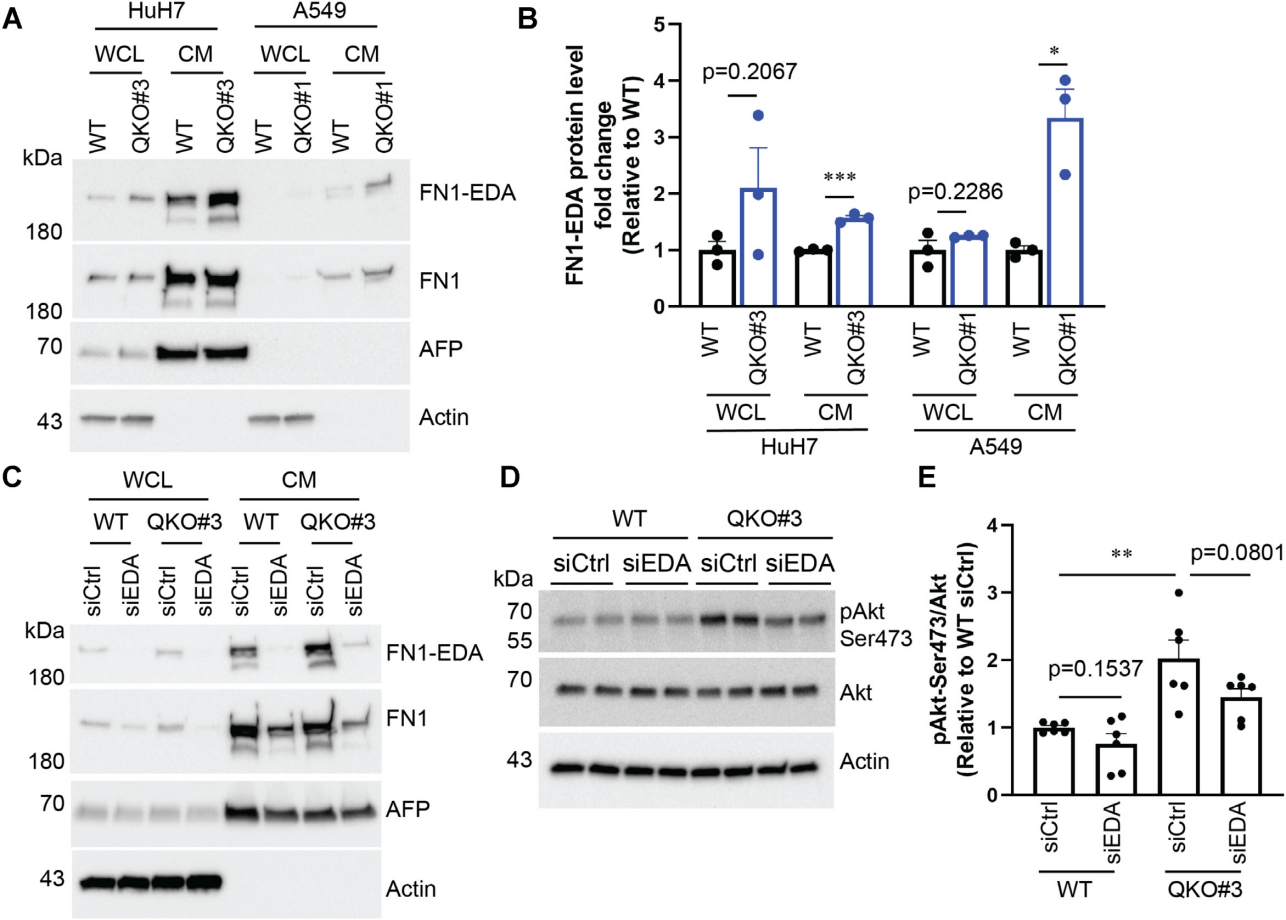
FN1-EDA protein expression is enhanced in QKO cells. (**A**, **B**) Cells were seeded in 12-well plates and on the following day, they were incubated with serum-free media for another 24hours. Whole cell lysates (WCL) and conditioned media (CM) were collected and probed for indicated proteins by immunoblotting. (**C–E**) Cells were transfected with two rounds of non-targeting siCtrl or siEDA. Approximately 24 h after the second siRNA transfection, transfected cells were serum-starved for another 24 h. WCL and CM were then collected and analyzed as mentioned above. Representative immunoblotting results were shown. Data are mean ± SEM from at least two independent experiments, and each experiment had one or two wells that were treated independently (replicates = 3 or 6). Each dot represents one biological replicate. Statistical significance was determined using a two-tailed *t* test: *P < 0.05; **P < 0.01, ***P < 0.001.

To test if increased inclusion of the EDA exon in *FN1* resulted in elevated FN1-EDA protein expression, we sought to determine FN1-EDA protein levels in both WT and QKO cells. Since FN1-EDA is detected in both cell lysates and supernatants (51,52), we probed for FN1-EDA in both whole cell lysates (WCL) and conditioned media (CM) using immunoblotting with an antibody specific to the amino acid sequence encoded by the EDA exon (Figure 4A). As a control, we also probed for FN1 with an antibody that recognizes all isoforms of FN1. The FN1-EDA signal was normalized to FN1, and then the data were presented relative to WT cells. The FN1-EDA protein level increased by around 50% in CM from HuH7 QKO#3 cells as compared with WT cells (Figure 4A and B). This increase in CM was not observed in FN1 or alpha fetoprotein (AFP), an abundant protein secreted by hepatocellular carcinoma cells. Though the FN1-EDA expression level in A549 is much lower than that in HuH7, QKI ablation similarly caused an increase in the FN1-EDA protein isoform (Figure 4A and B). Altogether, these data show increased expression of both cell-associated and secreted FN1-EDA protein in QKI-deficient cells, which is consistent with a higher level of EDA exon inclusion in FN1 transcripts.

Ectopic expression of FN1-EDA enhances activation of the Akt threonine/serine kinase, which can be assayed by detecting phosphorylation of serine 473 (53). Since QKI KO increases FN1-EDA protein expression, we conjectured that QKI could lead to Akt activation. Indeed, Akt serine 473 phosphorylation is increased ∼2-fold in QKO#3 as compared to WT cells (Figure 4C–E). To test if this Akt activation is dependent on FN1-EDA, we used an siRNA targeting sequences within the EDA exon (siEDA) to knockdown FN1-EDA and showed that it successfully reduced the expression of FN1-EDA (Figure 4C). FN1-EDA depletion in QKO#3 cells decreased phospho-Akt levels; however, this decrease was modest and did not reach statistical significance (Figure 4D and E). We reckon that increased Akt activation in QKO#3 cells is mediated by multiple factors, one of which is enhanced FN1-EDA protein expression (see discussion).

### QKI facilitates infectious viral particle production

**Figure 5.**
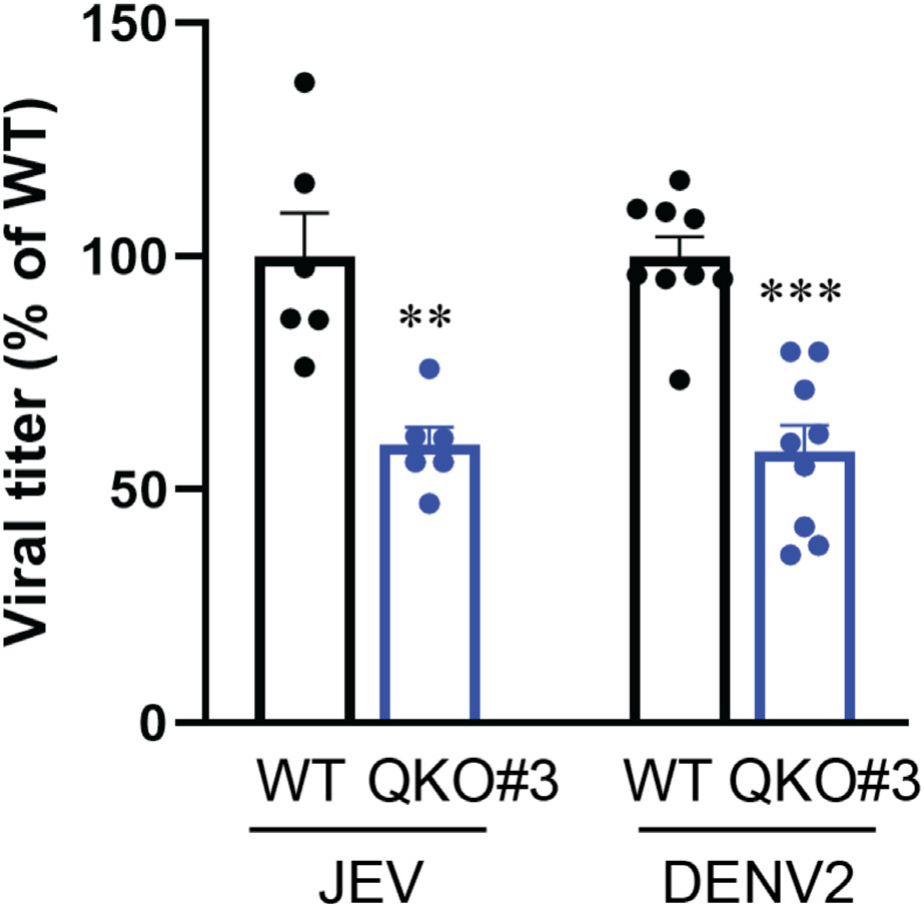
QKI promotes virus production. HuH7 WT and QKO#3 cells were infected with JEV at MOI = 0.5 and DENV2 at MOI = 10. At 24 h post-JEV infection and 48 h post-DENV2 infection, supernatants were collected and viral titer was determined by plaque assay. Data are mean ± SEM from at least two independent experiments, and each experiment had three wells that were treated independently (replicates = 6 or 9). Each dot represents one biological replicate. Statistical significance was determined using a two-tailed *t* test: **P < 0.01; ***P < 0.001.

Our data indicate that QKI can moderate the IFN system by down-regulating signaling via MAVS (26) (Figure 1 and Supplementary Figure S1) and reducing FN1-EDA proteins (Figures 2–4, Supplementary Figure S3). We hypothesized that QKI would facilitate viral replication because of its role in inhibiting host immunity. To test this hypothesis, we infected WT and QKO#3 cells with Japanese encephalitis virus (JEV) and Dengue virus serotype 2 (DENV2), both of which are flaviviruses (54). We then collected supernatants and measured viral titers to determine the consequence of QKI loss on infectious viral particle production. We observed about 50% reduction of both JEV and DENV2 infectious viral particles produced by QKO#3 cells (Figure 5). We conclude that QKI suppresses the IFN system via various pathways and subsequently facilitates viral replication (Supplementary Figure S3D).

## DISCUSSION

QKI is emerging as a critical immune suppressor. Our earlier study shows that QKI suppresses IFN response by downregulating MAVS protein expression (26). Additionally, QKI was shown to destabilize aryl hydrocarbon receptor mRNA and thus inhibit LPS-induced NFκB activation (24). In line with these observations, we demonstrated that QKI blocks inclusion of the EDA exon and higher levels of the FN1-EDA protein isoform accumulates in QKO cells. EDA has been shown to activate Toll-like receptor 4 (TLR4) pathways and promote TLR2 expression (31,55), which can ultimately lead to the production of pro-inflammatory cytokines. Additionally, our analysis of DEGs at the transcript level also sheds light into the potential molecular mechanisms by which QKI can inhibit the anti-viral IFN response. For example, both FSCN1 and GOLM1 RNA abundance are reduced in QKI-deficient cells. As both FSCN1 and GOLM1 were reported to inhibit IFN responses (44,45), we posit that knock-out of QKI leads to lower FSCN1 and GOLM1 expression and thus promotes host IFN response. Expression of either FSCN1 or GOLM1 alone, however, may not be sufficient to suppress enhanced IFN response in QKI deficient cells as QKI is likely to impact various aspects of innate immunity (24). Of note, dynamic changes in QKI expression upon stimulation correspond with its important role in regulating immune response. During early time points after LPS treatment, QKI expression is reduced (24,25), suggesting that cells need to derepress QKI inhibition to elicit robust immune response. At later time points, when the inflammatory response needs to be resolved, QKI expression is restored or increased to pre-stimulation level. In line with these predictions, prolonged treatment of IFN causes an increase of QKI expression at both RNA and protein levels (56), further suggesting a negative regulatory role of QKI in IFN signalling and immune response.

Alternative splicing is a key regulatory mechanism in controlling gene expression, and its role in innate immunity has been underappreciated until recently. We employed *vast-tools* to analyze our RNA-seq data and found that poly(I:C) triggered many alternative splicing events. More than half of these are IR events, suggesting that IR is a critical mechanism in regulating innate immunity. Indeed, IR has the ability to negatively regulate gene expression and control innate immunity by 1) delaying the onset of immune gene expression through slowing down splicing kinetics (57) and 2) increasing potential degradation of immune genes via non-sense mediated decay (NMD) (58). In line with these notions, retention of intron 4 in Irf7, a master IFN regulator, leads to reduced expression level of Irf7, potentially via NMD, and thus dampens Type 1 IFN response (59). Mechanistically, this retention of Irf7 intron 4 is controlled by an RNA-binding protein BUD13. BUD13 inhibits IR to promote Irf7 expression and thus positively regulate Type 1 IFN response and restrict viral replication. Interestingly, this IR event in Irf7 was observed at later time points of poly(I:C) stimulation (59), further confirming that IR can be a negative feedback regulatory mechanism of IFN response to avoid destructive immunity in cells. At nine hours post-transfection in our poly(I:C)-transfected cells, IR was observed in the STAT2 gene, a key transcription factor in IFN response. Although the function of this IR event in STAT2 remains to be characterized, it is plausible that IR in *STAT2* could provide a brake to tamp-down the immune response. Moreover, alternative splice site usage, coupled with transcript degradation, provides additional layers of regulation in modulating the IFN response against viral infection (60). In addition, we further examined the potential correlation between IR and transcript abundance in our data set and we found no correlation between them (Supplementary Table S4). While genes with decreased IR after poly(I:C) transfection were more likely to encode upregulated transcripts, (11 of 15; Supplementary Table S4), genes with increased IR upon poly(I:C) transfection or QKO, were evenly upregulated, downregulated or unchanged. Nevertheless, these results do not rule out the generality of such an association because our analysis was complicated by the strong stimulatory effect of poly(I:C) transfection at the transcriptional level.

Alteration of host splicing machinery by viral infection has also gained more attention recently (61,62). Comparison of dengue virus serotype 1 and its attenuated strain reveals the alteration of several alternative splicing events in host cells (63), which may result from a combination of processes taking place within infected cells. On the one hand, splicing changes can be a consequence of the cellular antiviral response. On the other hand, splicing alteration could be a proviral mechanism to counteract the antiviral response and to favor gene expression of proviral factors. Indeed, host immune genes were observed to undergo alternative splicing during DENV2 infection (64). In fact, several viral components have been shown to trigger splicing alteration in host cells. The DENV RNA dependent RNA polymerase NS5 influences cellular RNA splicing through the interaction of U5 snRNP core components CD2BP2 and DDX23 (64). Of particular interest is the ability of viral RNA alone to cause alteration in host splicing. Members of flaviviruses, ZIKV and DENV, produce copious amounts of non-coding subgenomic flaviviral RNA (sfRNA) during infection, and transfection of these sfRNAs alone is sufficient to cause aberrant splicing of SRSF7 by sequestering host splicing factor SF3B1 (65). Although the impact of these various alteration splicing events on viral replication remains to be fully characterized, a more comprehensive understanding of these pathways may provide potential avenues for therapeutic developments to treat infectious diseases.

In this study, we used a dsRNA analog, poly(I:C), to mimic viral infection and subsequently detected hundreds of differential splicing events. Though poly(I:C) can be detected by several RNA sensors (e.g. RIG-I), it is unclear if poly(I:C) sequesters other host proteins (e.g. splicing factors) to modulate host splicing machinery. In other words, poly(I:C)-triggered alternative splicing events could be a result of direct sequestration of host splicing factors by poly(I:C). Alternatively, the host splicing machinery could be altered as a consequence of dsRNA-induced host immunity. Obviously, these two processes are not mutually exclusive and further investigation is needed to delineate these mechanisms.

Spliced variants of FN1 which retain the extra domain A (EDA) exon have been associated with several human diseases (28–30,66,67), and thus alternative splicing of the EDA exon in FN1 must be tightly controlled. Earlier studies showed that exonic *cis*-acting elements in the EDA exon play essential roles in modulating EDA splicing (27). This regulatory mechanism, at least in part, is mediated by serine/arginine-rich (SR) proteins (e.g. SRSF1 and SRSF3) (68,69). Interestingly, transcription elongation rate and histone modification also contribute to the regulation of EDA exon splicing in FN1 (70). Here, we show that QKI is a novel *trans*-acting factor that promotes the exclusion of the EDA exon in FN1. We observed this regulatory mechanism in both human cells HuH7 and A549. This regulatory mechanism is conserved in mouse cells, as mouse Qk depletion caused an increase in the inclusion of EDA exon in Fn1. Mechanistically, our data suggest that QKI binds to the intron upstream of EDA exon and prevents its inclusion.

Akt is activated and phosphorylated in response to various environmental cues (71). We found that Akt phosphorylation increased in HuH7 QKO#3 cells. IFN alpha and beta treatment are known to activate Akt1 (72), so increased IFN signalling in HuH7 QKO#3 cells (26) can result in enhanced Akt phosphorylation in an autocrine manner. In addition, FN1-EDA activates Akt via the Toll-like receptor4 (TLR4) pathway (73). Thus, we tested if enhanced FN1-EDA expression also contributed to increased Akt phosphorylation. Intriguingly, we found that FN1-EDA is not absolutely required for activating Akt in HuH7 cells, and this lack of FN1-EDA dependency could be due to the following reasons. Firstly, HuH7 may not express all necessary TLR4 receptor complexes for responding to extracellular FN1-EDA (74,75). Secondly, FN1-EDA concentration in the culture media may not have been high enough to trigger TLR4 activation. Last, but not least, secreted full-length FN1-EDA requires enzymatic cleavage to expose cryptic sites for TLR4 agonization (76), which might not occur in our cell culture system. Altogether, our data show increased FN1-EDA protein secretion and Akt phosphorylation in HuH7 QKO#3 cells, both of which can contribute to the enhanced immune activation and IFN responses through paracrine- and autocrine-dependent pathways.

In conclusion, QKI plays many roles in controlling the host immune response. Here, we show that QKI is involved in the regulation of transcript levels for many genes that contribute to the IFN response. In addition, we demonstrate that QKI blocks the inclusion of the EDA exon in FN1. Elevated levels of FN1-EDA proteins secreted from QKI-deficient cells can contribute to immune activation and systemic inflammatory responses. It would be of great interest to investigate functional roles of QKI in regulating immune response at the whole organism level, and this line of research would provide additional insight into how QKI shapes host immunity.

## DATA AVAILBILIITY

RNA-seq data are available online under NCBI accessions PRJNA679633.

## Supplementary Material

gkab732_Supplemental_Files

## Notes

Present address: Kuo-Chieh Liao, Genome Institute of Singapore, A*STAR, Singapore.

Present address: Cassandra Modahl, Liverpool School of Tropical Medicine, UK.
