# Supplementary material for: The RNA binding protein Quaking represses splicing of the Fibronectin EDA exon and downregulates the interferon response
Source: Nucleic Acids Res. 2021 Aug 24;49(17):10034–45. doi: 10.1093/nar/gkab732 (PMC8464043; doi:10.1093/nar/gkab732)
Supplement: gkab732_Supplemental_Files [file gkab732_supplemental_files.zip › Liao et al Supplementary material_revised.docx]

**Supplemental Figure Legends**

**Figure S1. Analysis of differentially expressed genes (DEGs) between WT and QKO#3 cells.** (A) PCA analysis of RNA-seq data. (B) Venn diagrams showing DEGs between WT and QKO#3 cells with or without poly(I:C) transfection. (C) HuH7 WT and QKO#3 cells were left untreated or transfected with 1.5 μg poly(I:C). At nine hours post transfection, cellular RNA was isolated and IL6 transcript abundance was measured by RT-qPCR. The data were reported relative to the untransfected WT control. (D) Heatmap of DEGs in log2 scale. (E) Enrichment plots and heatmaps from the comparison with gene set “REACTOME_INTERFERON_GAMMA_SIGNALING”. Red and blue indicate up-regulated and down-regulated trends respectively. Data were reported relative to WT. Data are mean ± S.E.M from two independent experiments, and each experiment had two or three wells that were treated independently (replicates = 5 or 6). Each dot represents one biological replicate. Statistical significance was determined using a two-tailed *t* test: ***, p˂0.001.

**Figure S2. FSCN1 expression is regulated by QKI through its 3′UTR.**

(A) Representative immunoblotting results showing expression level of FSCN1 in WT and various QKO cells. Densitometry analysis was performed to quantify FSCN1 band intensity using ImageJ. Data were reported relative to WT. (B) eCLIP data showing regions bound to QKI-5. (C) HuH7 WT and QKO#3 cells were transfected with F Luc FSCN1 3′UTR and R Luc plasmids. One day post transfection, cells were harvested and luciferase activity was measured. Data are mean ± S.E.M from two independent experiments, and each experiment had two or three wells that were treated independently (replicates = 5 or 6). Each dot represents one biological replicate. Statistical significance was determined using a two-tailed *t* test: ***, p˂0.001. (D) Schematic illustration of QKI function in regulating host immunity.

**Figure S3. Modulation of splicing by QKI in the absence or presence of poly(I:C).** (A) Comparison of QKI-dependent splicing events with and without poly(I:C) transfection. (B) Heatmap showing differential splicing events. (C) HuH7 WT and QKO#3 cells were left untreated or transfected with 1.5 μg poly(I:C). At nine hours post transfection, lysates were processed and RT-PCR was performed to amplify splicing variants of indicated genes. PCR products were separated on 5% TAE-acrylamide gels or 1% agarose gel and imaged in a UV light transilluminator or analyzed using the Agilent 2100 Bioanalyzer. Representative data were shown. The ratio of inclusion to skipping was recorded as a percent of sequence inclusion (% Incl). Data are mean ± S.E.M from two independent experiments, and each experiment had three wells that were treated independently (replicates = 6). Exon/intron genomic coordinates are provided in Table S4. (D) Schematic illustration of QKI function in regulating host immunity.

**Figure S4. Conserved EDA regulatory splicing mechanism in mouse cell**. (A) Alignment of intron sequence upstream of EDA exon from different species. (B) Representative immunoblotting results showing Qk-5 expression in MEF cells and NIH/3T3 cells transfected with either non-targeting siRNA (siCtrl) or siQk. (C) MEF and NIH/3T3 cells were transfected with 50nM siCtrl or siQk and incubated at 37°C for two days before replacing with serum-free media. On the following day, RNA was isolated from cells and RT-PCR was performed to analyze mouse Fn1 splicing variants. PCR products were separated via TAE acrylamide gel electrophoresis or analyzed on the Agilent 2100 Bioanalyzer (representative shown). Percent of EDA sequence inclusion (% Incl) in the Fn1 splicing variants was reported. Data are mean ± S.E.M from two independent experiments, and each experiment had two or three wells that were treated independently (replicates = 4 or 5). (Statistical significance was determined using a two-tailed t test: **, p ˂ 0.01; ***, p ˂ 0.001.

**Supplemental materials and methods**

**Reporter assay**

FSCN1 3′ UTR (NM_003088.4) was amplified using forward primer

5′ - cgc gga tcc ggc cgg ccc gtc ctt ccc cgc - 3′ and reverse primer 5′ - cgc ctc gag tgg ggc tgc aga ctg agt tat - 3′ and digested with BamHI and XhoI restriction enzymes. Digested PCR products were subsequently cloned into a reporter construct to make Firefly luciferase (F Luc) FSCN1 3′ UTR plasmids. The inserted sequence was confirmed by Sanger sequencing. To perform the reporter assay, HuH7 WT and QKO#3 cells were seeded at 7.5x10^4^ cells per well in 24-well plates the day before transfection. Cells were co-transfected with 200ng F Luc FSCN1 3′ UTR reporter plasmids and 20ng *Renilla* luciferase (R Luc) plasmids using Lipofectamine 2000 (Thermo Fisher Scientific). On the following day, cells were lysed and luciferase activity was analyzed using the Dual Luciferase Reporter Assay kit (Promega) on a Promega plate reader. **Liao et al Supplementary material list**

**Table S1 DESeq2 results**

**Table S2 GSEA analysis**

**Table S3 Genotyping results**

**Table S4 *Vast-tools* results and comparison of splicing events**

**Table S5 Primers**
