# Supplementary figures and images for: The RNA binding protein Quaking represses splicing of the Fibronectin EDA exon and downregulates the interferon response
Source: Nucleic Acids Res. 2021 Aug 24;49(17):10034–45. doi: 10.1093/nar/gkab732 (PMC8464043; doi:10.1093/nar/gkab732)

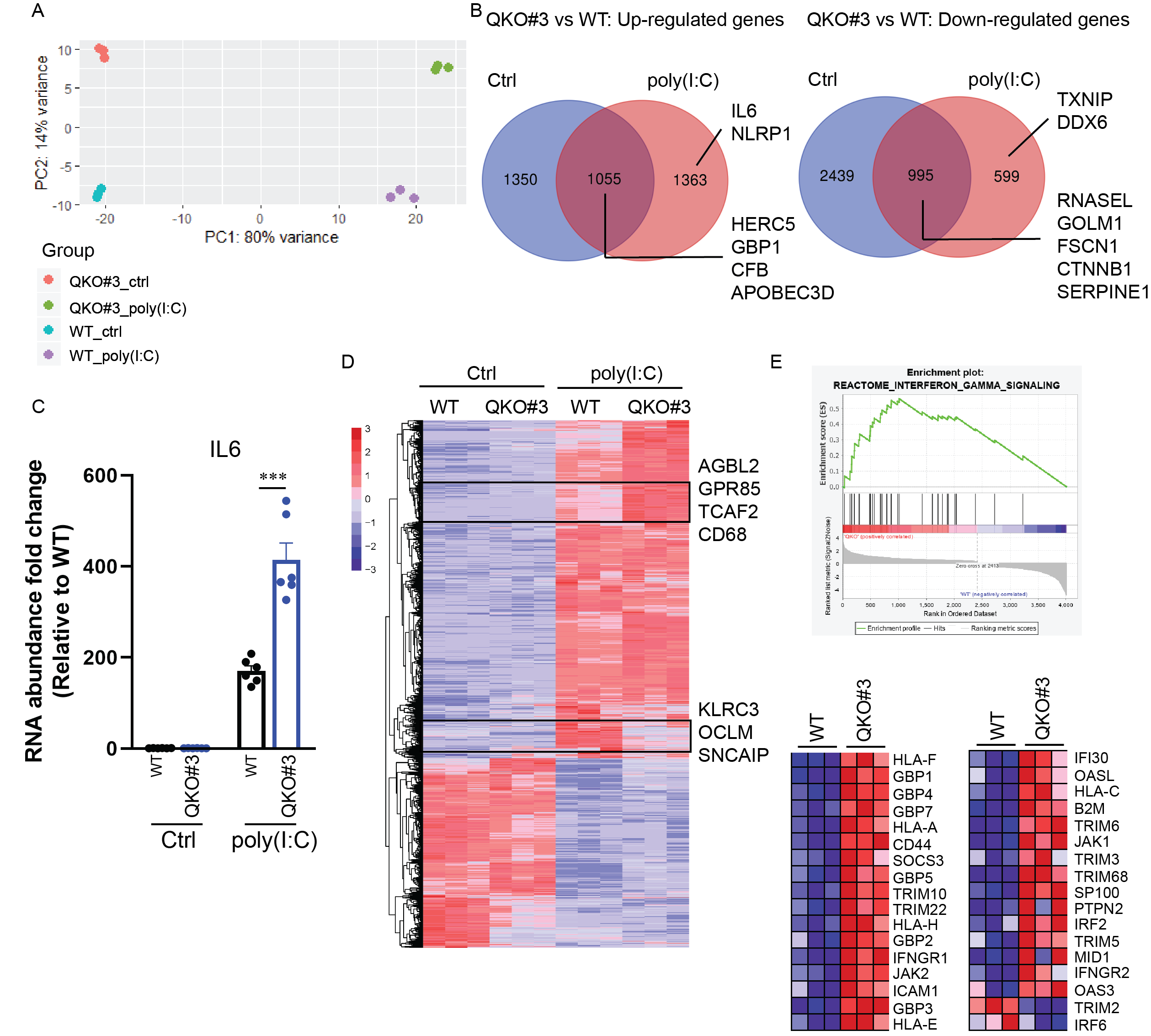

Supplement: gkab732_Supplemental_Files [file gkab732_supplemental_files.zip › FigureS1.png]

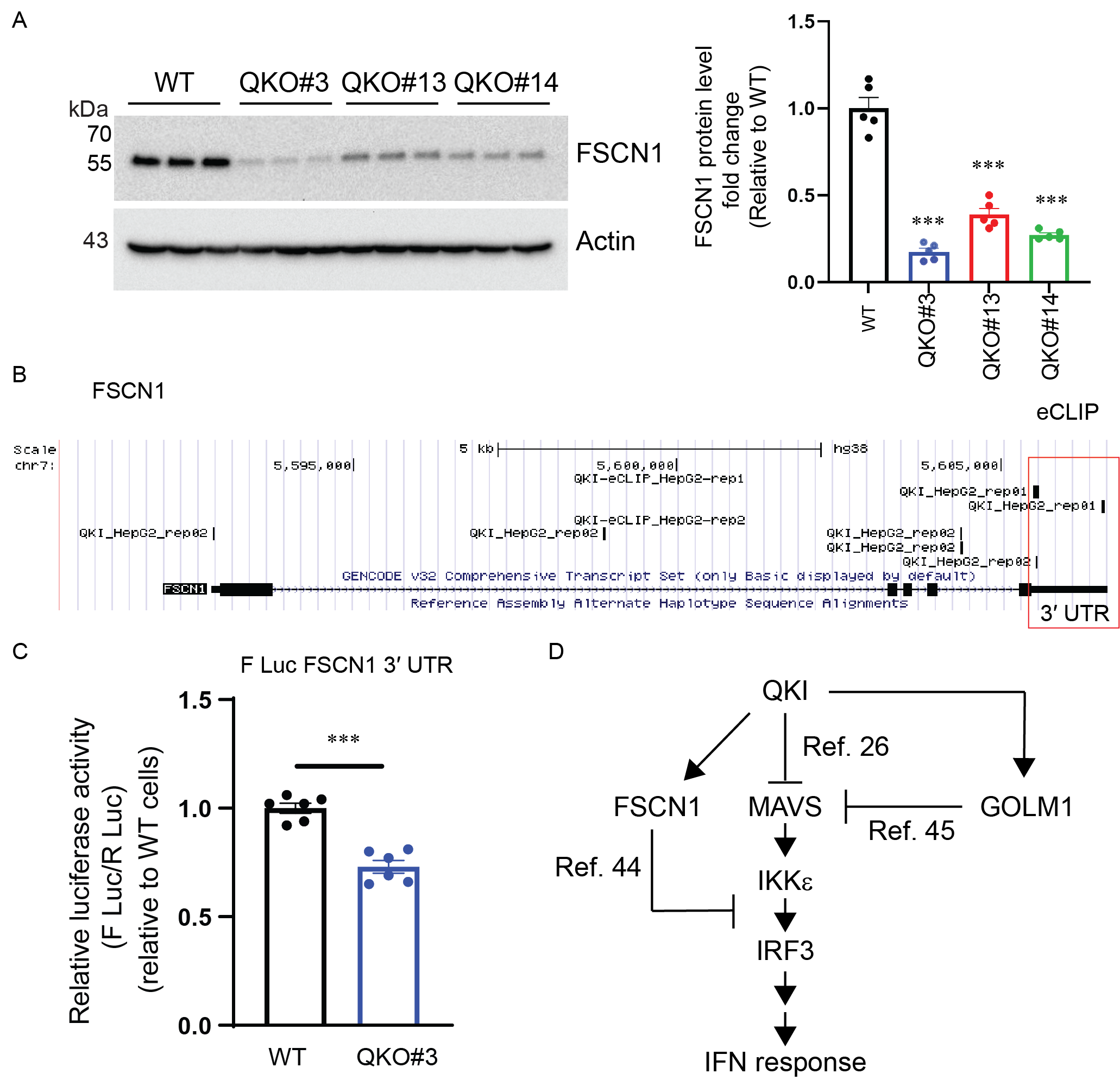

Supplement: gkab732_Supplemental_Files [file gkab732_supplemental_files.zip › FigureS2.png]

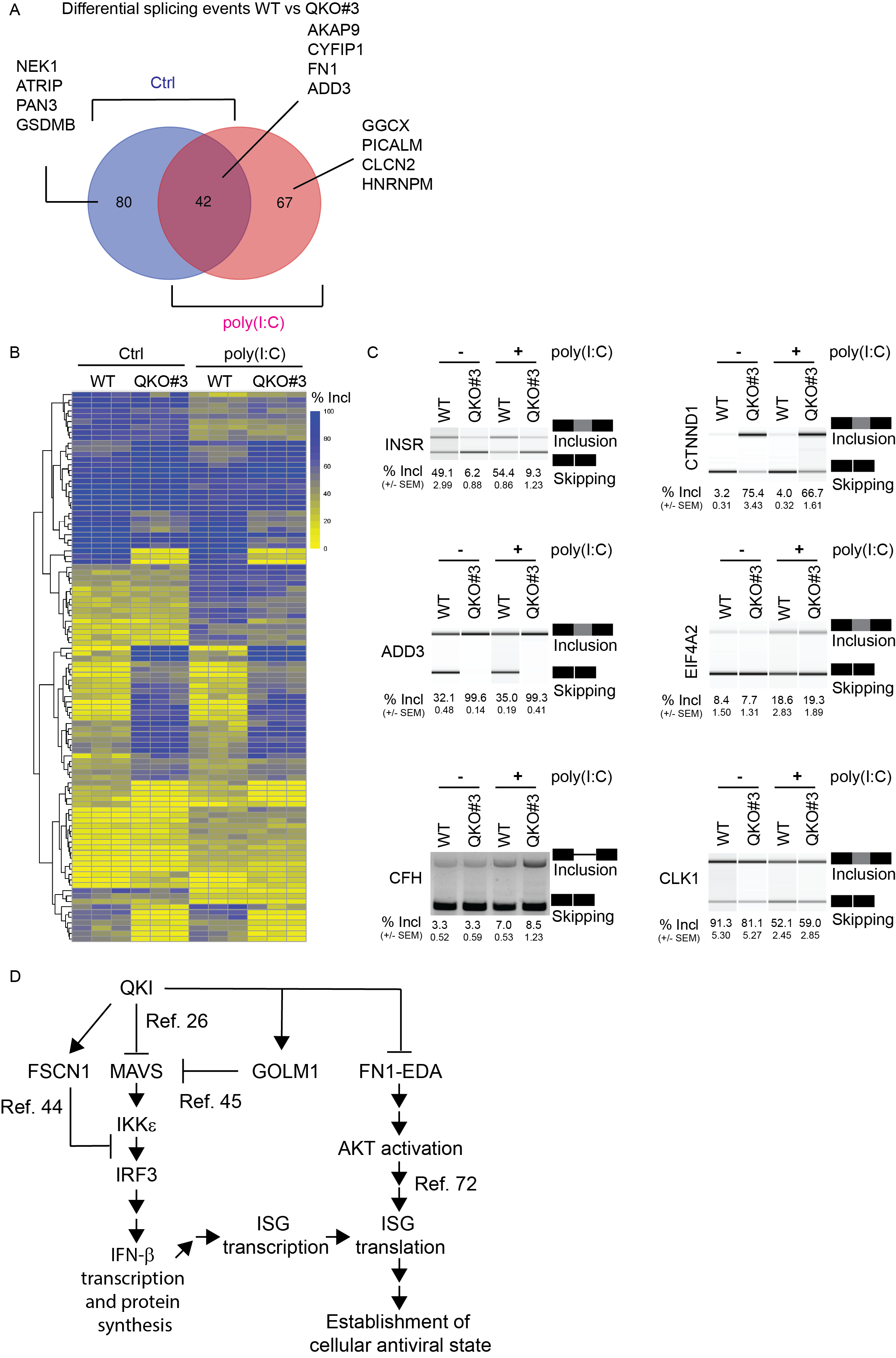

Supplement: gkab732_Supplemental_Files [file gkab732_supplemental_files.zip › FigureS3.png]

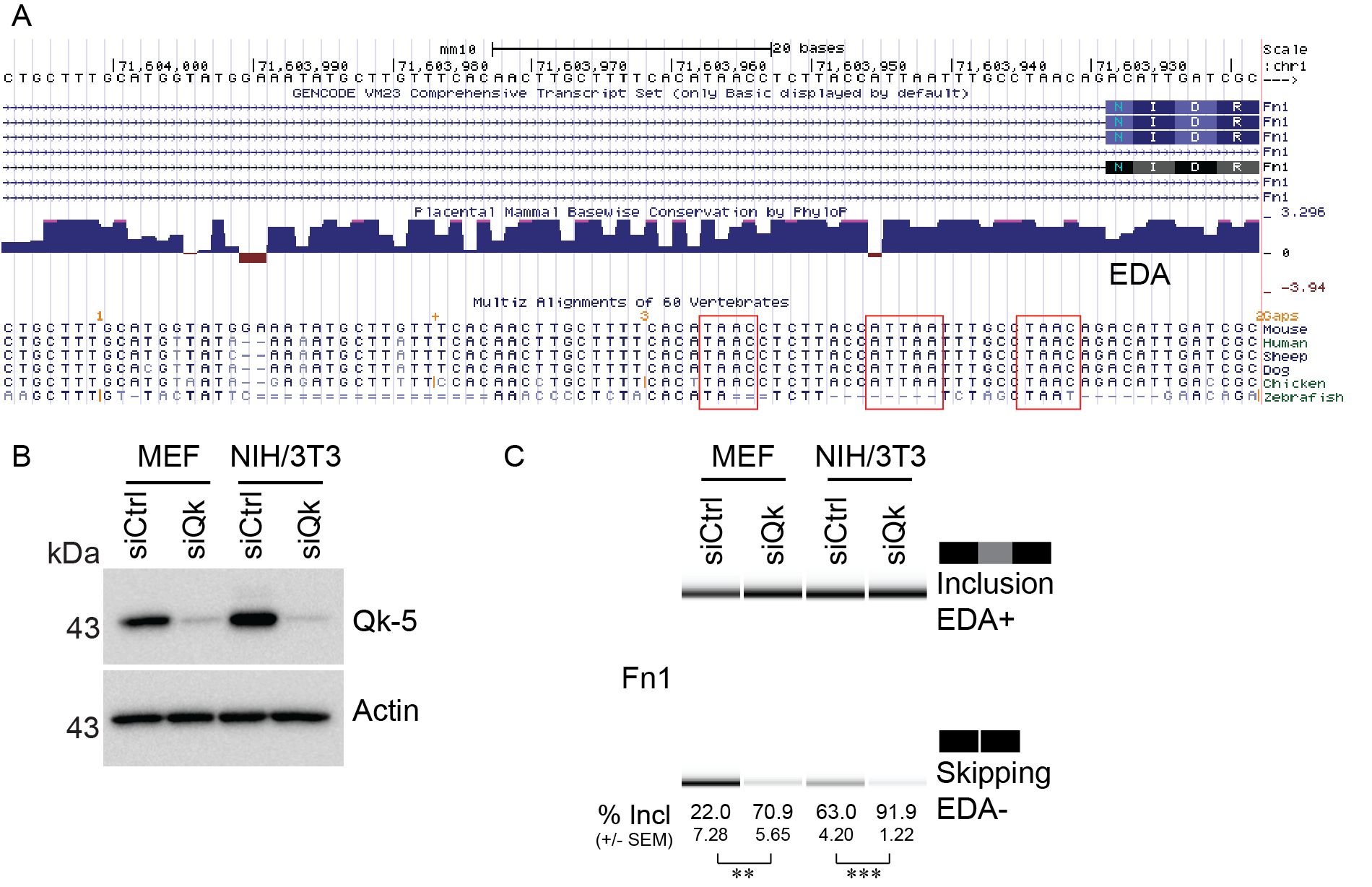

Supplement: gkab732_Supplemental_Files [file gkab732_supplemental_files.zip › FigureS4.png]
